# GATA3-Driven ceRNA Network in Lung Adenocarcinoma Bone Metastasis Progression and Therapeutic Implications

**DOI:** 10.3390/cancers17030559

**Published:** 2025-02-06

**Authors:** Yun Liu, Shihui Shen, Xudong Wang, Hansen Chen, Wenjie Ren, Haifeng Wei, Kun Li, Lei Li

**Affiliations:** 1 Shanghai Key Laboratory of Regulatory Biology, Institute of Biomedical Sciences, East China Normal University, Shanghai 200241, China; 2 Joint Center for Translational Medicine, Shanghai Fifth People’s Hospital, Fudan University and School of Life Science, East China Normal University, Shanghai 200240, China; 3 School of Life Sciences, East China Normal University, Shanghai 200241, China; 4 Department of Orthopedic Oncology, Changzheng Hospital, Shanghai 200003, China; 5 Department of Orthopedics, 905th Hospital of PLA Navy, Shanghai 200030, China; 6 Health Science Center, East China Normal University, Shanghai 200241, China

**Keywords:** lung adenocarcinoma bone metastasis, ceRNA network, NPRL3, GATA3, IL4R, E7449, dupilumab

## Abstract

**Background/Objectives**: Bone metastasis is a common and severe complication of lung adenocarcinoma (LUAD), impacting prognosis and treatment outcomes. Understanding the molecular mechanisms behind LUAD bone metastasis (LUADBM) is essential for developing new therapeutic strategies. The interactions between long non-coding RNAs (lncRNAs), microRNAs (miRNAs), and mRNAs in the competing endogenous RNA (ceRNA) network are crucial in cancer progression and metastasis, but the regulatory mechanisms in LUADBM remain unclear. **Methods**: Microarray analysis was performed on clinical samples, followed by weighted gene co-expression network analysis (WGCNA) and construction of a ceRNA network. Molecular mechanisms were validated using colony formation assays, transwell migration assays, wound healing assays to assess cell migration, and osteoclastogenesis assays to evaluate osteoclast differentiation. Potential therapeutic drugs and their binding affinities were predicted using the CMap database and K_deep_. The interaction between the small-molecule drug and its target protein was confirmed by surface plasmon resonance (SPR) and drug affinity responsive target stability (DARTS) assays. Mechanistic insights and therapeutic efficacy were further validated using patient-derived organoid (PDO) cultures, drug sensitivity assays, and in vivo drug treatments. **Results**: Our results identified the XLOC_006941/hsa-miR-543/NPRL3 axis as a key regulatory pathway in LUADBM. We also demonstrated that GATA3-driven Th2 cell infiltration creates an immunosuppressive microenvironment that promotes metastasis. Additionally, we confirmed that the inhibitor E7449 effectively targets NPRL3, and its combination with the IL4R-blocking antibody dupilumab resulted in improved therapeutic outcomes in LUADBM. **Conclusions**: These findings offer new insights into the molecular mechanisms of LUADBM and highlight potential therapeutic targets, including the XLOC_006941/miR-543/NPRL3 axis and GATA3-driven Th2 cell infiltration. The dual-target therapy combining E7449 with dupilumab shows promise for improving patient outcomes in LUADBM, warranting further clinical evaluation.

## Simple Summary

Bone is a common site of metastasis in lung adenocarcinoma, leading to poor prognosis and limited treatment options. This study analyzed gene expression data from clinical samples and identified the XLOC_006941/hsa-miR-543/NPRL3 axis as a key regulatory pathway in LUAD bone metastasis. Additionally, GATA3-driven Th2 cell infiltration was found to create an immunosuppressive microenvironment that promotes metastasis. The researchers demonstrated that the small molecule inhibitor E7449 effectively targeted NPRL3, inhibiting LUADBM patient-derived organoid growth and reducing metastasis in an in vivo mouse model. Furthermore, combining E7449 with the IL4R-blocking antibody dupilumab enhanced therapeutic outcomes. This dual-target therapy of E7449 and dupilumab shows promise for improving patient outcomes in LUADBM and warrants further clinical investigation.

## 1. Introduction

Lung adenocarcinoma (LUAD) is the most common subtype of non-small cell lung cancer (NSCLC), with bone metastasis affecting approximately 30% of patients with advanced-stage disease [1]. Bone metastasis leads to severe clinical complications, such as pain, fractures, and hypercalcemia, contributing to a poor prognosis [2]. Despite advances in cancer treatment, current therapies targeting bone metastasis in LUAD are inadequate, highlighting the urgent need for a deeper understanding of the underlying biological mechanisms involved.

Recent studies have elucidated the critical role of the ceRNA regulatory mechanism in tumor progression [3,4,5]. This mechanism is characterized by the competition between RNA transcripts for shared miRNA binding sites, thereby modulating the expression of target genes [5,6,7]. LncRNAs serve as miRNA sponges, sequestering miRNAs and preventing their binding to mRNAs, thus alleviating miRNA-mediated repression of gene expression. This interaction is crucial for the regulation of gene expression in a variety of cancers and other pathological conditions [8].

The bone microenvironment, composed of osteoblasts, osteoclasts, and various immune cells, forms a supportive stroma for metastatic tumor cells [9,10]. In LUAD, bone metastases are typically osteolytic, driven by a vicious cycle between tumor cells and hyperactivation of osteoclasts [11,12,13]. Tumor cells migrate to bone, inducing increased osteoclastic activity or disrupting the balance between osteoclastic resorption and bone formation, leading to localized or generalized bone loss. Osteoclast-mediated bone resorption plays a critical role in the invasion and metastasis of malignant cancer cells [14,15,16].

The immune-infiltrating microenvironment within bone plays a critical role in establishing the bone metastasis niche [17,18]. However, the specific contributions of immune cell populations, particularly T helper 2 (Th2) cells, remain poorly understood. Th2 cells have been shown to promote tumor progression through various immunosuppressive mechanisms [19,20,21]. Interleukin-4 (IL4), a key cytokine for Th2 differentiation, binds to IL4R, forming the type I IL4 receptor (IL4R-IL2RG) on lymphoid cells and the type II IL4 receptor (IL4R-IL13RA1) on non-lymphoid and tumor cells. IL4 not only drives Th2 differentiation but also stimulates tumor cell proliferation and metastasis [22,23,24]. While these mechanisms have been explored in other primary tumors, the role of Th2 cells and the IL4R axis in LUADBM remains unexplored.

This study investigates gene expression profiles associated with LUADBM, focusing on the regulatory role of ceRNA networks. We identified key ceRNA axes, including the XLOC_006941/hsa-miR-543/NPRL3 pathway, which promote LUADBM progression. Additionally, we observed a Th2-shifted immune microenvironment that supports the development of LUADBM. We explored therapeutic strategies targeting these pathways, such as the NPRL3 inhibitor E7449, which shows promise for LUADBM treatment. Furthermore, combining anti-IL4R antibodies with E7449 enhances therapeutic efficacy in PDO models of LUADBM. Our findings provide new insights into the genetic and immune regulation of LUADBM and highlight potential therapeutic strategies to improve patient outcomes.

## 2. Materials and Methods

### 2.1. Lung Adenocarcinoma Bone Metastasis Clinical Specimens

Clinical tissue specimens were collected from Shanghai Changzheng Hospital, including paired fresh tissues of normal lung (approximately 5 cm away from the tumor site) and lung adenocarcinoma from the primary LUAD patients (*n* = 10) and fresh bone metastasis tissues from LUADBM patients (*n* = 10). Bone metastasis in the clinical samples was confirmed by PET/CT imaging and immunohistochemistry. The study was approved by the Research Ethics Committee of East China Normal University (HR 123-2022). The clinical details of these patients are included in Supplementary Table S1.

### 2.2. Differential Expression Analysis of lncRNAs, miRNAs, and mRNAs

Raw counts of lncRNAs, miRNAs, and mRNAs were normalized using the trimmed mean of M values (TMM) method. Principal component analysis (PCA) was performed to evaluate sample distribution and identify potential outliers. Differentially expressed genes were identified based on a *p*-value < 0.05 and fold change ≥2 or ≤0.5, with statistical significance determined using the Wald test and adjusted for false discovery rate (FDR) via the Benjamini–Hochberg procedure. The DESeq2 package (v1.38.3) in R software (v4.2.2) was employed for the analysis.

### 2.3. Weighted Gene Co-Expression Network Analysis

Weighted gene co-expression network analysis (WGCNA) is a systems biology method used to identify co-expression modules and hub genes based on the correlation between genes. Pre-processing of raw data involved removing missing values, quantile normalization, and filtering using the WGCNA package (v1.72-5) in R software. The soft threshold power was set to 22 based on the scale-free topology criterion. Sample dendrogram and trait heatmap were plotted based on lncRNA and mRNA expression data and clinical traits. To ensure that each sample was in a different cluster, revealing the distribution of clinical data, the algorithm approach was utilized. Co-expression modules were identified using the dynamic tree cut algorithm with a minimum module size of 30. The association between module eigengenes and clinical traits was evaluated by calculating the Pearson correlation coefficients to identify the modules associated with clinical traits. The hub genes and lncRNAs within the co-expression modules were identified based on their connectivity with other genes in the module. The genes with high module membership values (>0.9) and high gene significance values (>0.9) were considered hub genes or lncRNAs.

### 2.4. CeRNA Network Construction

The ceRNA network was constructed using 3862 lncRNAs and 4924 mRNAs derived from WGCNA module 2, which was identified as the module most strongly associated with LUADBM. To refine this gene set and focus on the most functionally relevant genes in LUADBM, we applied a weighted threshold of 0.35, in addition to cutoffs for gene significance (GS) and module membership (MM) of 0.9. This approach resulted in a final gene set comprising 203 lncRNAs and 242 mRNAs. Furthermore, we included 49 downregulated miRNAs in LUADBM relative to LUAD tissues. To identify co-regulated lncRNA-miRNA and miRNA-mRNA pairs, we employed bioinformatics tools such as DIANA-LncBase (V3), miRDB, starBase (v2.0), and miRWalk (2.0). The interactions were visualized using the ggplot2 package (v3.5.1) in R software.

### 2.5. Osteoclastogenesis

Mouse bone marrow-derived monocytes (BMMs) were isolated from mouse tibia and cultured in α-MEM medium with 10% FBS and M-CSF (30 ng/mL) (PeproTech, Cranbury, NJ, USA) for 24 h. The lower layer of cells was digested and seeded into a 96-well plate with α-MEM medium containing 10% FBS, M-CSF (30 ng/mL), RANKL (50 ng/mL) (R&D Systems, Minneapolis, MN, USA), and conditioned medium from cultured A549L6 cells. The medium was refreshed every other day. After 5–7 days, BMMs differentiated into mature osteoclasts, which were observed microscopically. Tartrate-resistant acid phosphatase (TRAP) staining was performed using the TRAP kit (Sigma-Aldrich, St. Louis, MO, USA) to assess TRAP activity. Osteoclast numbers were quantified as multinucleated TRAP-positive cells per unit length.

### 2.6. Surface Plasmon Resonance

Surface plasmon resonance (SPR) experiments were conducted using a Biacore T200 system (GE Healthcare Life Sciences, Chicago, IL, USA). Recombinant NPRL3 protein was purified and diluted in acetate buffer (pH 7.5), then immobilized onto the CM5 Biacore sensor chip through an EDC/NHS cross-linking reaction to achieve a target immobilization level of 25 RU. E7449, prepared in running buffer containing 5% DMSO, was injected at concentrations ranging from 1 to 16 μM into both the reference and NPRL3 channels at a flow rate of 30 μL/min, with each injection and dissociation lasting 120 s. Sensorgrams obtained from the Biacore T200 were analyzed using Biacore T200 evaluation software, and data were fitted to the steady-state affinity model (1:1) to calculate the equilibrium dissociation constant (K_d_).

### 2.7. Organoid Culture and Drug Sensitivity Assessment

During the pre-treatment phase, fresh patient-derived organoid samples are washed with a cleaning solution, then sliced into small fragments and incubated at 37 °C for 30 min in digestive fluid. After red blood cell lysis and resuspension, cell counts are performed using AO/PI staining. The cells are then centrifuged at 1500 rpm for 5 min, dispensed into a matrix-coated 48-well plate with AEBL medium, and incubated at 37 °C for 10–15 min. For drug sensitivity evaluation, organoids are added to 96-well plates once they reach a size of 30–100 μm. After 96 h of exposure, PDO viability is assessed using calCIN-AM/PI fluorescence staining, and organoid activity is observed via microscopy.

### 2.8. Cell Lines and Cell Culture

In this study, we utilized two human LUAD cell lines A549 and H441 and one mouse Lewis lung carcinoma cell line LLC1 that were procured from the American Type Culture Collection (ATCC). The cells were cultured in Dulbecco’s Modified Eagle’s Medium (DMEM) (Gibco, Grand Island, NY, USA) supplemented with 10% fetal bovine serum (FBS) (Vazyme, Nanjing, China) and 1% penicillin/streptomycin (Vazyme, Nanjing, China) at 37 °C in a humidified atmosphere containing 5% CO_2_. Subculturing of the cells was carried out every three to four days using 0.25% trypsin-EDTA solution (Thermo Fisher Scientific, Waltham, MA, USA), and the cell count was determined using a hemocytometer. For subsequent assays, the cells were seeded at a density of 5 × 10^4^ cells/well in 6-well plates or 1 × 10^5^ cells/well in 12-well plates and allowed to attach overnight before being subjected to further treatment.

### 2.9. Western Blot

Cells and tissues were homogenized in RIPA buffer (Beyotime, Shanghai, China), and protein concentrations were measured using the BCA protein assay (Beyotime, CN). A total of 20 μg of protein was subjected to 10% SDS-PAGE gels, then transferred to a PVDF membrane (Millipore, Burlington, MA, USA), followed by 30 min of 5% milk blocking and overnight primary antibody incubation at 4 °C. After horseradish peroxidase-conjugated secondary antibody incubation, visualization was performed on the Oddesey Western blotting system and software (LI-COR Biosciences, Lincoln, NE, USA). Primary antibodies directed against IL4R (1:2000, Proteintech, Rosemont, IL, USA), NPRL3 (1:1000, CUSABIO Life Sciences, College Park, MD, USA), GATA3 (1:2000, CST, Kansas City, MO, USA), STAT6 (1:2000, Proteintech, USA), p-STAT6 (1:2000, CST, USA), β-actin (1:5000, MBL, Japan), and GAPDH (1:2000, Proteintech, USA) were used. GAPDH or β-actin was used as the internal control.

### 2.10. RNA Extraction and RT-qPCR

Total RNA extraction was performed using TRIzol reagent (Invitrogen, Waltham, MA, USA) following the manufacturer’s instructions. The quality and quantity of the RNA were assessed using a NanoDrop ND-1000 spectrophotometer (Thermo Fisher Scientific, USA). Subsequently, cDNA was synthesized from 1 μg of total RNA using the PrimeScript RT reagent kit (TaKaRa, Tokyo, Japan).

To quantify gene expression levels, we performed quantitative real-time PCR (RT-qPCR) using the SYBR Premix Ex Taq II kit (TaKaRa, Tokyo, Japan) on a 7500 Real-Time PCR System (Applied Biosystems, Waltham, MA, USA). The RT-qPCR reaction mixture included 10 μL of 2× SYBR Premix Ex Taq II (Vazyme, Nanjing, China), 0.4 μL of each primer (10 μM), 0.4 μL of ROX Reference Dye II (50×), 2 μL of cDNA template, and 6.8 μL of ddH_2_O. The cycling conditions for RT-qPCR were set as follows: 95 °C for 30 s, followed by 40 cycles of 95 °C for 5 s and 60 °C for 34 s. We computed the relative expression levels of the target genes using the 2^−ΔΔCt^ method and normalized them to GAPDH expression.

### 2.11. Molecular Docking

The human NPRL3 cryo-electron microscopy structure model (PDB code: 6CES) was used for molecular docking simulation. The docking of small molecule E7449 against the NPRL3 model was performed using Schrödinger Suite 2022. The LigPrep program with the OPLS3 force field was employed to generate and refine the initial 3D configurations of the small molecule. Default settings were used for processing the protein structures of NPRL3. Molecular docking was then performed with the Glide program in an extra precision mode (Glide XP), and the most favorable pose was selected for further analysis.

### 2.12. Constructs and Reagents

To achieve knockdown of human genes, annealed sense and antisense shRNA oligonucleotides were cloned into the pLKO.1 vector (Addgene, Watertown, MA, USA). For overexpression of genes, the full length of the gene mRNA sequence was constructed in the pLVX vector. To analyze binding activity, the full length of lncRNA or mRNA 3′UTR was cloned into the psiCHECK-2 vector (Promega, Madison, WI, USA).

### 2.13. Colony Formation Assay

Cells were seeded at a density of 2000 cells per well in 12-well plates and incubated for 24 h to allow cell attachment. The cells were then treated with various concentrations of the test compound for 24 h. After exposure, the cells were washed with PBS and cultured in fresh medium for an additional 10–14 days to allow colony formation. The medium was changed every 3–4 days during this time. After the incubation period, colonies were fixed with 4% paraformaldehyde (Sangon Biotech, Shanghai, China) and stained with 0.1% crystal violet (Beyotime, Shanghai, China). Colonies consisting of more than 50 cells were counted manually using a light microscope. Survival fractions were calculated as the ratio of the number of colonies formed in treated versus control wells.

### 2.14. Wound Healing Assay

Stable cells were inoculated in a 6-well plate at an appropriate density to achieve an 80% confluent monolayer the following day. A standardized scratch wound was created using a 10 µL pipette tip, and phase-contrast images were taken at 0 h and 24 h from the same location in the petri dish to monitor wound closure. The cell migration rate was calculated and analyzed using ImageJ 1.54 software, while the degree of wound closure was quantified by measuring the distance between the edges of the scratch wound.

### 2.15. Transwell Migration Assay

Cells were cultured in an appropriate medium until they reached the desired level of confluency. Subsequently, the cells were resuspended and counted using serum-free medium, and 5 × 10^4^ cells were seeded into the upper chamber of a transwell with 200 μL of serum-free medium. The lower chamber was added with 500 μL of full medium containing 10% fetal bovine serum. The transwell was incubated at 37 °C temperature and 5% CO_2_ concentration for 24 h. After incubation, the cells from the upper surface of the membrane were wiped off slightly using a cotton swab, while the cells on the lower surface of the membrane were fixed with 4% paraformaldehyde and stained with 0.1% crystal violet. The stained cells were visualized under a microscope, and the number of migrating cells through the membrane was counted.

### 2.16. Dual Luciferase Reporter Assay

The plasmids were created by incorporating the DNA sequence of interest containing the WT/Mut binding site of miRNA into the psiCHECK-2 reporter vector upstream of the Renilla luciferase gene. In addition, a constitutively expressed firefly luciferase gene was included in the vector. The cells were cultured until they reached 70–80% confluency in a 24-well plate, and then the constructed plasmids and pGMLV miRNA plasmid were co-transfected using the Lipo8000 transfection reagent. After incubation at 37 °C temperature and 5% CO_2_ concentration for 24–48 h, the cells were lysed with passive lysis buffer according to the manufacturer’s protocol. Separate luciferase assays were performed for firefly and Renilla luciferase activity using a dual luciferase reporter assay kit, and luminescence was measured using a luminometer. Three independent experiments were conducted, and data analysis involved normalizing the Renilla luciferase signal to the firefly luciferase signal to correct for differences in transfection efficiency and background noise.

### 2.17. Prediction of Small Molecule Drug Candidates

To identify potential drugs capable of reversing the expression of genes most strongly associated with LUADBM, we imported the top 150 upregulated and downregulated mRNAs into the Connectivity Map (CMap) database and utilized enrichment score ranking to identify the top five small-molecule compounds or drugs. To ascertain the binding affinity between these small molecules and the seven mRNAs present in the final ceRNA network, we utilized an established online tool known as K_deep_, a pretrained machine learning model based on deep convolutional neural networks that predicts protein-ligand binding affinity. The computed pK_i_ scores provided insight into the interaction between small molecules and proteins.

### 2.18. Drug Affinity Responsive Target Stability Assay

A549L6 cells were cultured to approximately 80–85% confluence, rinsed with ice-cold PBS, and lysed with lysis buffer (150 mM NaCl, 1.0% NP-40, 0.5% sodium deoxycholate, 50 mM Tris, pH 8.0). Cells were harvested and lysed for 10 min on ice. Cells were centrifuged at 18,000× *g* for 20 min at 4 °C to collect the supernatant. Protein concentration was determined via BCA assay. Ten μM E7449, 30 μM E7449, or 1 μL DMSO were incubated with aliquots of cell lysate at 5 mg/mL for 30 min with shaking at room temperature. Pronase (Sigma-Aldrich, St. Louis, MO, USA) was added to 20 μL aliquots of cell lysates at 0.01 μg/μL for 15 min at room temperature. Digestion was stopped by adding a 1× protease inhibitor cocktail (Sigma-Aldrich, St. Louis, MO, USA) and incubating the reactions on ice for 10 min. SDS-PAGE loading buffer (6 μL of 5×) was added to the samples, and samples were heated for 10 min at 100 °C. Finally, samples were resolved by SDS-PAGE, and the degradation of NPRL3 was analyzed by Western blot.

### 2.19. Cell Viability Assay

Transfected A549L6 cells were collected and adjusted to the proper concentration and then put in 96-well plates with 5 × 10^3^ cells in each well. When the cells grew adhering to the wall in an incubator with 5% CO_2_ at 37 °C for 24 h, the E7449 group was added with the drug-containing medium for 48 h, with the final concentration of E7449 as 0, 0.015, 0.045, 0.136, 0.410, 1.230, 3.700, 11.110, 33.330, and 100 µmol/L, respectively. Then, after the supernatant was carefully removed, 110 µL of CCK8 solution (100 µL free medium and 10 µL CCK8) was added, and the incubation continued at 37 °C for 1 h, and then 100 µL of working fluid was added to terminate the reaction. After leaving it at room temperature for 20 min until purple granules were dissolved, the absorbance at the wavelength of 450 nm was measured by a microplate reader. Drug concentration at the cell growth inhibition rate of 50% (IC_50_) was calculated by GraphPad.

### 2.20. LUADBM Mouse Model and Therapy

The in vivo model utilized male, 6-week-old BALB/c nude mice obtained from the Animal Center of East China Normal University and raised under pathogen-free conditions. The study was conducted in accordance with national and institutional guidelines and approved by the Institutional Animal Care and Use Committee.

To initiate LUADBM, 1 × 10^6^ viable A549L6 cells transfected with luciferase were injected into the left ventricle of each BALB/c nude mouse (*n* = 5 for each group). Bone lesions caused by bone metastasis were monitored weekly using the Caliper IVIS Lumina II system (PerkinElmer, Shelton, CT, USA). After 5 weeks post-injection, the affected bones were collected, fixed in 4% paraformaldehyde for 24 h, and subjected to further μCT analysis.

Male C57BL/6 mice aged 6–8 weeks were randomly divided into three groups, each consisting of 5 mice. The control group received no treatment, while the other two groups were injected in situ with 5 × 10^6^ LLC1 cells in the lung and tibia, respectively, to model lung adenocarcinoma or bone metastasis. After 2 weeks, the mice were euthanized by cervical dislocation, and the tumor tissues were extracted from the injection sites. The tissues were processed into single-cell suspensions and analyzed using flow cytometry to compare the immune microenvironment across the different groups.

In the in vivo administration of E7449, following the formation of the bone metastasis model over a period of two weeks, E7449 was dissolved in 5% carboxymethylcellulose and administered intragastrically to mice at a dose of 40 mg/kg (*n* = 5), once daily for 21 days. Bioluminescence imaging was conducted weekly to monitor bone destruction.

### 2.21. Flow Cytometric Analysis

To obtain a single-cell suspension of tumor cells, mice were euthanized, and their tumors were removed and placed in a dish with 1–2 mL of cold PBS on ice. The tumors were minced into small pieces, then 1 mL of digestion buffer (10 U/mL Collagenase I, 100 U/mL Collagenase IV, 30 U/mL DNase I in PBS) was added and mixed. The tissue was digested at 37 °C for 30 min, passed through a 70 µm filter, and gently ground to obtain single cells. After centrifuging at 1500 rpm for 10 min and discarding the supernatant, the cells were treated with red blood cell lysis buffer for 2 min. They were then filtered again, washed, and resuspended in PBS with 2% FBS. The suspension was blocked with anti-CD16/32 antibody for 20 min at 4 °C, followed by staining with antibodies (CD3ε/FITC, CD4/PerCP-Cy5.5, IL4/PE-Cy7, IFNγ/PE (BioLegend, San Diego, CA, USA)) for 30 min in the dark. After washing, the cells were resuspended in PBS and analyzed by flow cytometry using an LSR Fortessa, with data processed in FlowJo (Tree Star, Woodburn, Oregon, USA).

### 2.22. Statistical Analysis

The statistical analysis was performed using R software version 4.2.2 or GraphPad Prism 8.0. All statistical tests were two-sided, and *p* < 0.05 was considered statistically significant. Continuous variables were presented as mean ± s.e.m. A Student’s *t*-test (unpaired, two-tailed) or one-way ANOVA was used to compare means between two or multiple groups of continuous variables. Spearman’s rank test was employed to determine the correlations.

## 3. Results

### 3.1. lncRNA XLOC_006941 Promotes LUADBM Initiation and Metastasis via a Novel ceRNA Network

**Figure 1:**
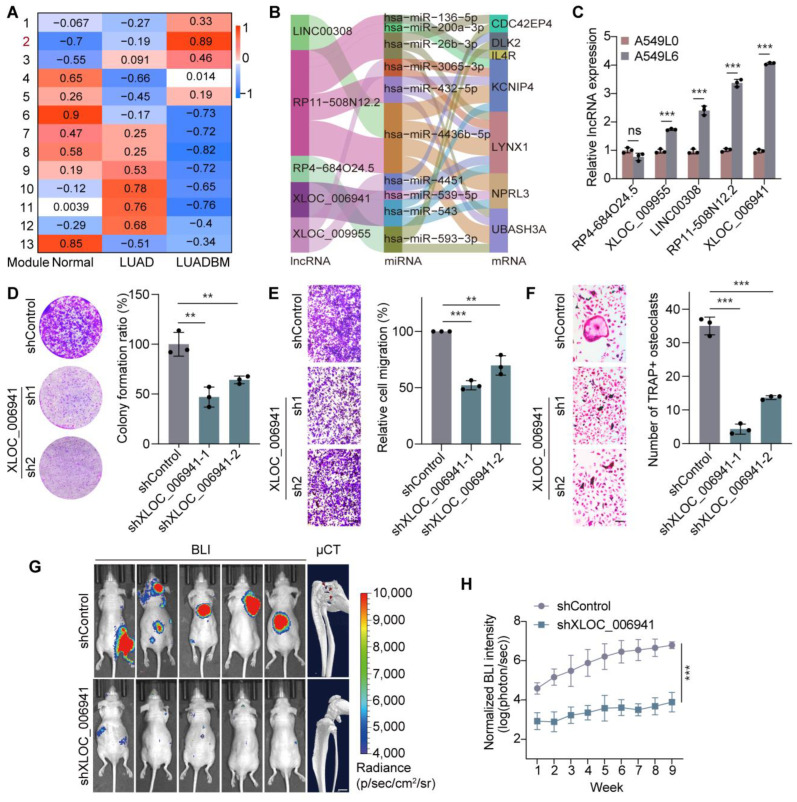
lncRNA XLOC_006941 promotes LUADBM initiation and metastasis via a novel ceRNA network. (**A**) Heatmap showing lncRNA and mRNA expression categorization into modules and their correlations with clinical traits; (**B**) Sankey diagram illustrating the ceRNA network and potential molecule interactions; (**C**) RT-qPCR quantification of five lncRNAs in the ceRNA network between A549L0 and A549L6 cells; (**D**) Colony formation assay (left) and bar graph (right) depicting colony numbers in control and XLOC_006941 knockdown cell lines; (**E**) Transwell assay images (left) and bar graph (right) showing migration differences in control and XLOC_006941 knockdown cells. Scale bar, 200 µm; (**F**) Osteoclastogenesis ability in BMMs between control and XLOC_006941 knockdown cells (left, images; right, bar graph). Scale bar, 200 µm; (**G**) Bioluminescence (BLI, left) and μCT (right) images showing the effect of XLOC_006941 knockdown in a mouse model of bone metastasis, with red arrows indicating bone destruction. Scale bar, 1 mm; (**H**) Weekly quantification of BLI intensity in mice receiving shXLOC_006941 or control H441 cells. Data shown represent mean ± s.e.m. (*n* = 3). ns, not statistically significant, ** *p* < 0.01, *** *p* < 0.001; *p* values were analyzed by an unpaired, two-tailed *t*-test or one-way ANOVA multiple comparisons test.

In this study, we analyzed microarray data from 30 clinical specimens, including 10 paired samples of adjacent normal tissues and LUAD tissues from patients with primary LUAD, as well as 10 bone metastasis tissues from LUADBM patients. To explore the regulatory mechanisms in LUADBM, we performed gene co-expression network analysis (WGCNA) to identify lncRNA and mRNA co-expression modules across normal lung, LUAD, and LUADBM tissues. WGCNA identified thirteen modules, where genes within the same module are likely to function cooperatively. Module 2 exhibited the strongest association with LUADBM (Figure 1A and Figure S1A). This module contained 3862 lncRNAs and 4924 mRNAs, showing the strongest correlation between gene significance and membership in module 2 (Figure S1B).

lncRNAs function as molecular sponges for miRNAs, modulating mRNA expression by sequestering miRNAs [25,26,27,28]. Based on this, we constructed a ceRNA network using interactions from non-coding RNA databases [29,30,31,32], identifying 5 key lncRNAs, 10 miRNAs, and 7 mRNAs as central components of the network (Figure 1B). The expression levels of the five lncRNAs progressively increased from normal lung tissues to LUAD, and further to LUADBM tissues (Figure S1C). To validate the expression of these lncRNAs, we utilized A549L6 cells, a LUADBM cell line derived from A549L0 cells through three rounds of bone metastasis induction by left ventricular injection into mice [33]. Four out of the five lncRNAs were upregulated in A549L6 cells compared to A549L0 cells (Figure 1C), with XLOC_006941 showing the most significant upregulation.

To investigate the specific function of the ceRNA network in LUADBM progression, we focused on XLOC_006941. shRNA-mediated knockdown of XLOC_006941 significantly reduced the colony-forming ability of H441 LUAD metastasis cells (Figure 1D and Figure S1D). Additionally, knockdown of XLOC_006941 impaired cell migration (Figure 1E and Figure S1E). These results highlight the critical role of XLOC_006941 in promoting LUAD cell proliferation and migration.

Bone metastasis is characterized by bone destruction driven by osteoclast activity [2,34]. To explore the role of XLOC_006941 in this process, we isolated bone marrow macrophages (BMMs) from mouse tibia, which differentiate into mature osteoclasts upon stimulation with macrophage colony-stimulating factor (M-CSF) and receptor activator of nuclear factor kappa-B ligand (RANKL). Conditioned medium (CM) from H441 cells with stable knockdown of XLOC_006941 significantly inhibited osteoclast differentiation when combined with RANKL and M-CSF, as confirmed by tartrate-resistant acid phosphatase (TRAP) staining (Figure 1F).

To assess the in vivo role of XLOC_006941 in bone metastasis, we utilized a left ventricle injection mouse model and found that suppression of XLOC_006941 significantly reduced bone metastasis (Figure 1G). Weekly BLI confirmed a consistent decrease in metastatic burden, highlighting the role of XLOC_006941 in LUADBM-induced bone destruction (Figure 1H). These results suggest that XLOC_006941 plays a critical role in LUADBM progression by promoting tumor cell proliferation, migration, and osteoclast-driven bone destruction, thereby contributing to the bone metastasis phenotype.

### 3.2. hsa-miR-543 and NPRL3 as Downstream Molecules Regulated by XLOC_006941 Function in the Progression of LUADBM

**Figure 2:**
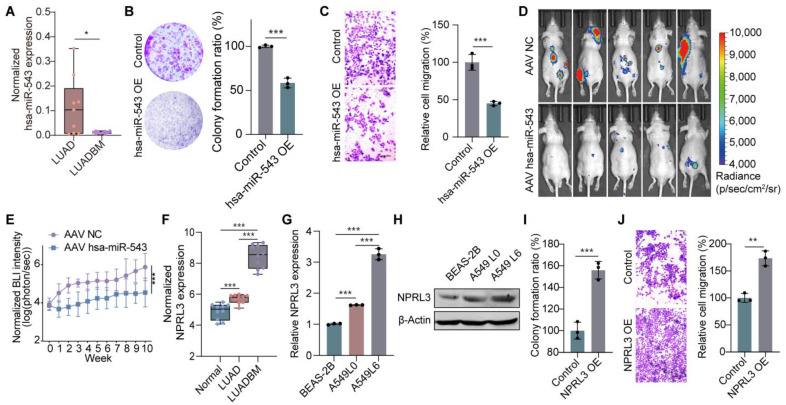
hsa-miR-543 and NPRL3 as downstream molecules regulated by XLOC_006941 function in the progression of LUADBM. (**A**) Normalized hsa-miR-543 expression in LUAD and LUADBM microarray data; (**B**) Colony formation images (left) and statistical analysis (right) comparing hsa-miR-543 overexpression (OE) cells to controls; (**C**) Transwell assay images (left) and bar graph (right) of control and hsa-miR-543 overexpression cells. Scale bar, 200 µm; (**D**) BLI showing the effect of AAV negative control (NC) and AAV hsa-miR-543 in a LUADBM mouse model induced by H441 left ventricular injection; (**E**) Weekly variation in AAV hsa-miR-543 treatment compared to AAV NC via tail vein injection in a LUADBM mouse model induced by H441 left ventricular injection; (**F**) Normalized NPRL3 expression in normal, LUAD, and LUADBM tissues; (**G**) Relative NPRL3 RNA expression in BEAS-2B, A549L0, and A549L6 cells; (**H**) NPRL3 protein expression in BEAS-2B, A549L0, and A549L6 cells; (**I**) Colony formation ratios comparing NPRL3 overexpression cells to controls; (**J**) Transwell assay images (left) and bar graph (right) of control and NPRL3 overexpression cells. Scale bar, 200 µm. Data shown represent mean ± s.e.m. (*n* = 3). * *p* < 0.05, ** *p* < 0.01, *** *p* < 0.001; *p* values were analyzed by the unpaired, two-tailed *t*-test or one-way ANOVA multiple comparisons test.

Subcellular localization analysis revealed that XLOC_006941 is present in both the cytoplasm and nucleus of LUADBM cells (Figure S2A), suggesting its potential cytoplasmic function as a ceRNA by sponging miRNA [35]. We investigated the regulatory mechanisms of XLOC_006941 in the LUADBM ceRNA network and hypothesized that XLOC_006941 may regulate NPRL3 expression through interaction with hsa-miR-543 or hsa-miR-539-5p (Figure 1B). Knockdown of XLOC_006941 led to the upregulation of hsa-miR-543, but not hsa-miR-539-5p (Figure S2B). Additionally, hsa-miR-543 expression was lower in LUADBM tissues compared to LUAD tissues (Figure 2A), suggesting that hsa-miR-543 functions as a sponge miRNA of XLOC_006941.

We further explored the role of hsa-miR-543 in LUADBM. Overexpression of hsa-miR-543 in H441 cells significantly inhibited cell proliferation (Figure 2B and Figure S2C) and migration (Figure 2C and Figure S2D). In a LUADBM mouse model, H441 cells were injected into the left ventricle to induce bone metastasis, followed by tail vein injection of AAV encoding hsa-miR-543. BLI and μCT analysis revealed that AAV-mediated delivery of hsa-miR-543 significantly reduced bone metastasis compared to the AAV-negative control (Figure 2D,E).

Among the mRNAs regulated downstream of XLOC_006941, NPRL3 was identified as a key target. NPRL3 expression was most significantly downregulated in cells with XLOC_006941 knockdown (Figure S2E). We also observed a stepwise increase in NPRL3 expression from normal lung tissue to LUAD and further to LUADBM tissues (Figure 2F). NPRL3 mRNA and protein levels were higher in A549L0 cells, with a further increase in A549L6 cells compared to BEAS-2B cells (Figure 2G,H). NPRL3 overexpression promoted cell proliferation and migration (Figure 2I,J and Figure S2F–H). Together, these findings highlight the critical roles of hsa-miR-543 and NPRL3 within the ceRNA axis, demonstrating their involvement in the progression of LUADBM.

### 3.3. XLOC_006941 Sponges hsa-miR-543 to Rescue NPRL3, Forming a ceRNA Network That Regulates LUADBM Progression

**Figure 3:**
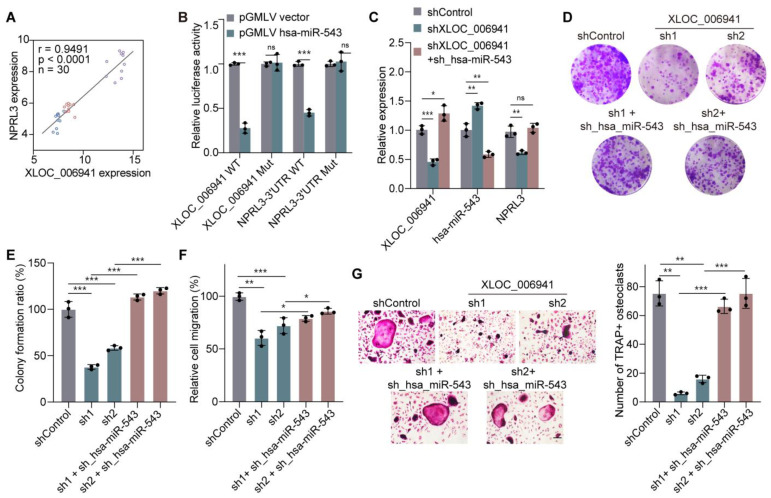
XLOC_006941 sponges hsa-miR-543 to rescue NPRL3, forming a ceRNA network that regulates LUADBM progression. (**A**) Expression correlation between XLOC_006941 and NPRL3 in LUADBM microarray data; (**B**) Relative luciferase activity displays the interaction between XLOC_006941 WT/Mut or NPRL3 3′UTR WT/Mut and hsa-miR-543. WT, wild type; Mut, mutant; (**C**) Relative expression in XLOC_006941 knockdown and double knockdown of XLOC_006941, hsa-miR-543 cells; (**D**) Representative images of colony formation in XLOC_006941 knockdown and XLOC_006941, hsa-miR-543 double knockdown cells; (**E**) S Statistical analysis of colony formation in XLOC_006941 knockdown and double knockdown cells; (**F**) Wound healing assay results after 24 h; (**G**) BMM osteoclastogenesis assay results after 7 days, with representative images (left) and statistical analysis (right). Scale bar, 200 µm. Data shown represent mean ± s.e.m. (*n* = 3). ns, not statistically significant, * *p* < 0.05, ** *p* < 0.01, *** *p* < 0.001; *p* values were analyzed by the unpaired, two-tailed *t*-test or one-way ANOVA multiple comparisons test.

A strong positive correlation was observed between the expression levels of XLOC_006941 and NPRL3 (Figure 3A), indicating that XLOC_006941 positively regulates NPRL3. Using the RNAhybrid algorithm [36], we identified potential binding sites for hsa-miR-543 within XLOC_006941 and within the 3′UTR of NPRL3 (Figure S3A). Dual luciferase reporter assays confirmed the functional interaction, showing that hsa-miR-543 binding reduced luciferase activity for both XLOC_006941 and NPRL3. This reduction in luciferase activity was abolished upon mutation of the predicted binding sites (Figure 3B), validating the ceRNA regulatory mechanism involving the XLOC_006941/hsa-miR-543/NPRL3 axis.

In the ceRNA model, reduced lncRNA expression can increase free miRNAs, which subsequently repress target mRNAs [37]. To test this hypothesis, we suppressed hsa-miR-543 in XLOC_006941 knockdown cells, which restored the expression of both XLOC_006941 and NPRL3 (Figure 3C). This rescue resulted in restored colony formation capacity (Figure 3D,E), partial recovery of migration defects (Figure 3F and Figure S3B), and a partial rescue of osteoclast differentiation, which had been impaired in XLOC_006941 knockdown cells (Figure 3G).

These findings highlight the critical role of the XLOC_006941/hsa-miR-543/NPRL3 ceRNA network in regulating osteoclast-driven bone destruction and LUADBM progression.

### 3.4. GATA3 Functions as a Transcription Factor of the ceRNA Network in LUADBM

**Figure 4:**
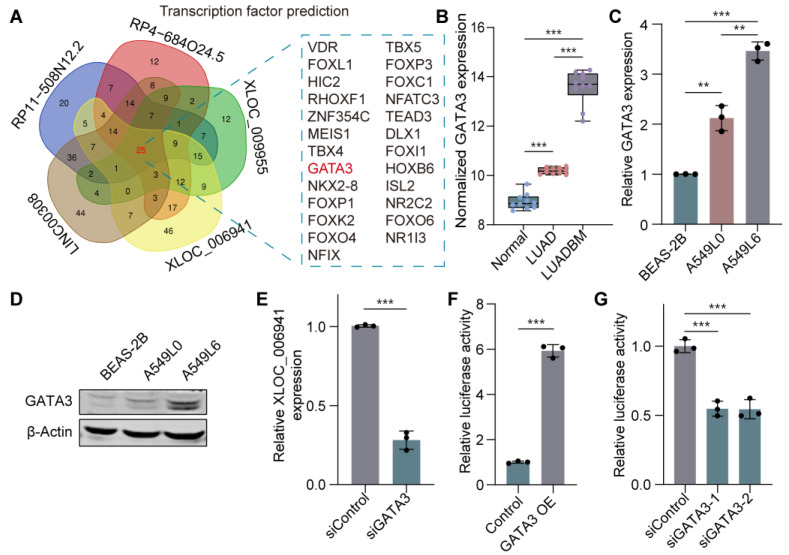
GATA3 functions as a transcription factor of the ceRNA network in LUADBM. (**A**) Venn diagram illustrating the number of transcription factors potentially binding to motifs within five lncRNAs, analyzed using the JASPAR database; (**B**) Normalized GATA3 expression in microarray data from normal, LUAD, and LUADBM tissues; (**C**) Relative RNA expression of GATA3 in A549L0 and A549L6 cells compared to BEAS-2B cells; (**D**) Protein expression of GATA3 in BEAS-2B, A549L0, and A549L6 cells; (**E**) Relative XLOC_006941 expression in GATA3 knockdown cells compared to control cells; (**F**) Relative luciferase activity of XLOC_006941 promoter reporter in A549L6 cells with or without GATA3 overexpression; (**G**) Relative luciferase activity of XLOC_006941 promoter reporter in A549L6 cells with or without GATA3 knockdown. Data shown represent mean ± s.e.m. (*n* = 3). ** *p* < 0.01, *** *p* < 0.001; *p* values were analyzed by the unpaired, two-tailed *t*-test or one-way ANOVA multiple comparisons test.

Then, we aimed to investigate the regulatory mechanisms underlying the complex ceRNA network in LUADBM. Previous studies suggest that lncRNAs are primarily regulated at the transcriptional level by transcription factors (TFs) binding to their promoter regions [38,39,40]. To explore this, we utilized the JASPAR database [41] and identified 25 transcription factors potentially involved in regulating the ceRNA network in LUADBM (Figure 4A). Among these, GATA3 was significantly upregulated in LUADBM compared to LUAD (fold change of 10.415) and in LUAD compared to normal tissues (fold change of 2.379) (Figure 4B). Notably, a GATA3-binding motif (AGATAA) was present in the promoter regions of all five key lncRNAs associated with the ceRNA network (Figure S4A,B), suggesting potential direct transcriptional regulation by GATA3.

Further validation in cell lines revealed that GATA3 expression was significantly elevated at both the mRNA and protein levels in A549L6 cells compared to A549L0 and BEAS-2B cells (Figure 4C,D). To assess the functional role of GATA3 in regulating lncRNAs, we performed siRNA-mediated knockdown of GATA3 (Figure S4C), which resulted in a notable reduction in the expression of the lncRNA XLOC_006941 (Figure 4E). Luciferase reporter assays confirmed that overexpression of GATA3 significantly enhanced the promoter activity of XLOC_006941 (Figure 4F and Figure S4D). Conversely, GATA3 knockdown reduced the promoter activity of XLOC_006941 (Figure 4G and Figure S4E). These results collectively establish GATA3 as a key transcriptional regulator of the identified lncRNAs in LUADBM and emphasize its role in promoting tumor progression through transcriptional regulation of the ceRNA network.

### 3.5. Increased Th2 Cell Infiltration and a GATA3/RP11-508N12.2/hsa-miR-136-5p/IL4R ceRNA Axis Contribute to LUADBM Progression

**Figure 5:**
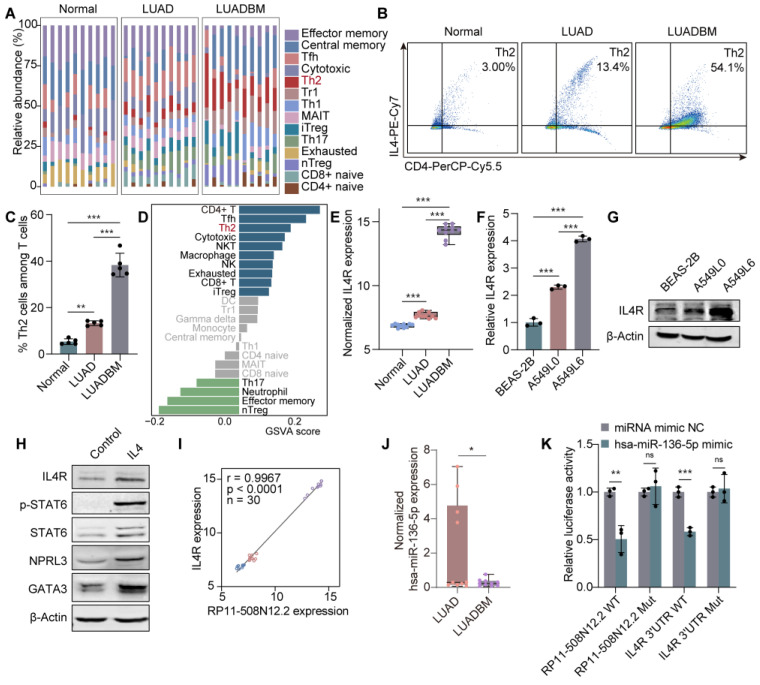
Increased Th2 cell infiltration and a GATA3/RP11-508N12.2/hsa-miR-136-5p/IL4R ceRNA axis contribute to LUADBM progression. (**A**) Immune cell abundance in LUADBM; (**B**) Representative flow cytometry plots of Th2 cells within T cells in normal, LUAD, and LUADBM tissues; (**C**) Percentages of Th2 cells within T cells in normal, LUAD, and LUADBM tissues; (**D**) GSVA analysis of the 7 mRNAs in the ceRNA network using the immune dataset; (**E**) Normalized IL4R expression in LUAD and LUADBM tissues; (**F**) IL4R RNA expression in BEAS-2B, A549L0, and A549L6 cells; (**G**) IL4R protein expression in BEAS-2B, A549L0, and A549L6 cells; (**H**) Protein expression following 200 ng/mL IL4 treatment for 30 min in A549L6 cells; (**I**) Correlation between RP11-508N12.2 and IL4R in LUADBM microarray data; (**J**) Normalized expression of hsa-miR-136-5p in LUAD and LUADBM tissues; (**K**) R Luciferase activity showing interaction between RP11-508N12.2 WT/Mut or IL4R 3′UTR WT/Mut and hsa-miR-136-5p. WT, wild type; Mut, mutant. Data shown represent mean ± s.e.m. (*n* = 3). ns, not statistically significant, * *p* < 0.05, ** *p* < 0.01, *** *p* < 0.001; *p* values were analyzed by the unpaired, two-tailed *t*-test or one-way ANOVA multiple comparisons test.

Given that GATA3 is a key transcription factor in Th2 cell differentiation [42,43], and IL4R, a surface marker of Th2 cells [44,45,46], was identified as a central mRNA in the ceRNA network in LUADBM (Figure 1B), we investigated the potential role of Th2 cells in LUADBM through the functions of GATA3 and IL4R. Immune microenvironment analysis using the ImmuCellAI tool [35] revealed a significant increase in the Th2 cell subset in LUADBM tissues compared to LUAD and normal tissues (Figure 5A). Elevated expression levels of Th2 markers, especially IL4R and GATA3, were detected in LUADBM tissues (Figure S5A). Single-cell RNA sequencing data from the Gene Expression Omnibus (GEO) database (accession: GSE123902) [47] further confirmed a progressive increase in Th2 markers from normal to LUAD and LUADBM tissues (Figure S5B,C).

To further investigate the role of Th2 cells in LUADBM, we established an in vivo mouse model by injecting LLC1 cells into the lung to induce LUAD and into the tibia to model LUADBM. After 4 weeks, flow cytometry analysis revealed a higher abundance of Th2 cells in both LUAD and LUADBM groups compared to control tissues (Figure 5B,C and Figure S5D). Correlation analysis revealed significant associations between the expression of these lncRNAs and Th2 cell infiltration scores (Figure S5E). Gene set variation analysis (GSVA) of the mRNAs in the ceRNA network also revealed increased abundance of Th2 cells (Figure 5D). These findings indicate that Th2 polarization is a prominent feature of the LUADBM immune microenvironment.

IL4R, a Th2 marker, was progressively upregulated in LUADBM tissues (Figure 5E) and in A549L0 and A549L6 cells compared to BEAS-2B cells at both the mRNA and protein levels (Figure 5F,G). IL4, secreted by Th2 cells, binds to IL4R on tumor cells, leading to activation of signal transducer and activator of transcription 6 (STAT6) and the subsequent upregulation of GATA3 and IL4 expression [48]. Interestingly, IL13RA1 expression did not significantly change in LUADBM tissues (Figure S5F), suggesting that IL4R, rather than IL13RA1, plays a functional role in the receptor complex. In A549L6 cells, IL4 treatment induced STAT6 activation, increased phosphorylation, and upregulated the expression of GATA3, IL4R, and NPRL3 (Figure 5H).

CeRNA analysis identified IL4R as a downstream target of lncRNA RP11-508N12.2, which is upregulated in A549L6 cells compared to A549L0 cells and regulates multiple mRNAs through miRNAs (Figure 1B,C). Moreover, RP11-508N12.2 and IL4R were found to compete for binding to hsa-miR-136-5p, supporting a regulatory feedback loop. A strong positive correlation between RP11-508N12.2 and IL4R expression was observed, further suggesting a regulatory relationship between these two molecules (Figure 5I). hsa-miR-136-5p was identified as a key miRNA linking RP11-508N12.2 to IL4R, with a downregulation of hsa-miR-136-5p observed in LUADBM tissues (Figure 5J). Sequence analysis identified binding sites between RP11-508N12.2 and hsa-miR-136-5p, as well as between the 3′UTR of IL4R and hsa-miR-136-5p (Figure S5G). Luciferase reporter assays confirmed the direct interaction (Figure 5K).

These findings emphasize a regulatory network involving GATA3 and IL4R, crucial for Th2 cell differentiation, and highlight the significance of Th2 polarization in the LUADBM microenvironment.

### 3.6. Targeting the ceRNA Network Offers Promising Therapeutic Strategies for LUADBM

**Figure 6:**
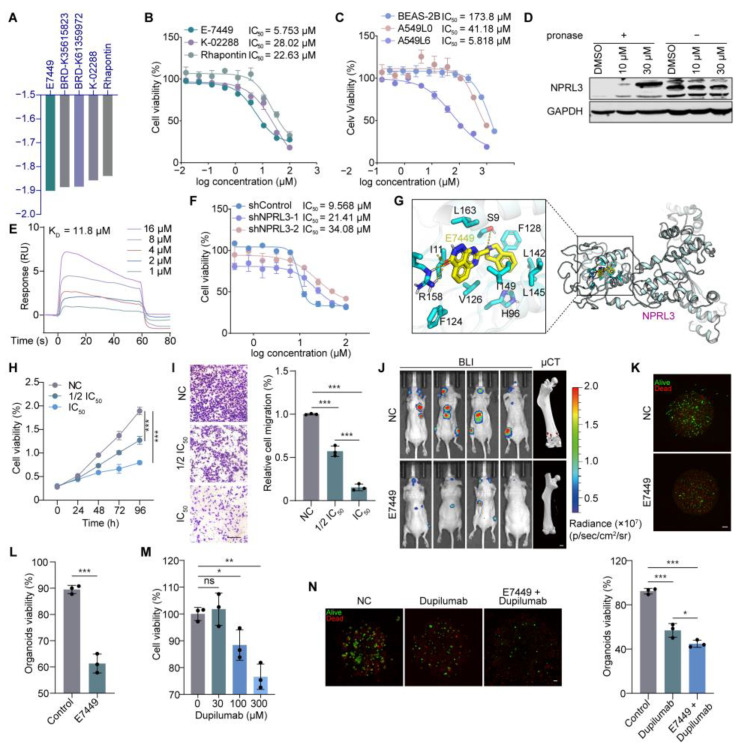
Targeting the ceRNA network offers promising therapeutic strategies for LUADBM. (**A**) The top five small molecule drugs predicted by the CMap database to potentially reverse gene expression in LUADBM; (**B**) IC_50_ values of small molecules E7449, K-02288, and Rhapontin in A549L6 cells; (**C**) IC_50_ values of E7449 in BEAS-2B, A549L0, and A549L6 cells; (**D**) NPRL3 expression in DARTs assay following treatments with DMSO, 10 µM E7449, or 30 µM E7449; (**E**) SPR sensorgrams showing the binding of E7449 to immobilized NPRL3; (**F**) IC_50_ values of E7449 in control and NPRL3 knockdown A549L6 cells; (**G**) Docking model of E7449 with NPRL3 N-terminal longin domain; (**H**) A549L6 cell viability following treatment with 1/2 IC_50_ or IC_50_ concentration of E7449 over 96 h; (**I**) Representative images (left) and statistical bar plot (right) comparing the migration of control cells and E7449-treated cells by the transwell assay. Scale bar, 200 µm; (**J**) BLI image (left) and µCT (right) showing the effect of 40 mg/kg E7449 in a mouse model of LUADBM induced by H441 left ventricular injection. Red arrows in the μCT image highlight the regions of bone destruction. Scale bar, 1 mm; (**K**) Representative fluorescent images of LUADBM PDO treated with 5 µM E7449; (**L**) Statistic bar plot of LUADBM PDO treated with 5 µM E7449; (**M**) Cell viability after treatment with varying concentrations of dupilumab, followed by a 30-min induction with 200 ng/mL IL4; (**N**) Representative fluorescent images of LUADBM PDO treated with 150 µg/mL dupilumab or a combination of E7449 and dupilumab (left) and corresponding bar plot (right). Scale bar, 200 µm. Data shown represent mean ± s.e.m. (*n* = 3). ns, not statistically significant, * *p* < 0.05, ** *p* < 0.01, *** *p* < 0.001; *p* values were analyzed by the unpaired, two-tailed *t*-test or one-way ANOVA multiple comparisons test.

To explore potential therapeutic strategies targeting the dysregulated ceRNA network in LUADBM, we employed the Connectivity Map (CMap) to identify small molecules that could reverse aberrant gene expression [49]. From the top 150 upregulated and downregulated genes in the LUADBM-related module 2 identified by WGCNA, we selected five compounds with the highest connectivity scores: E7449, BRD-K35615823, BRD-K61359972, K-02288, and Rhapontin (Figure 6A). Using K_deep_ [50], a deep learning tool for predicting protein-ligand binding affinities, we evaluated the binding potential of these compounds. E7449 exhibited strong binding affinities (pK_i_ > 8) for KCNIP4, LYNX1, and NPRL3, suggesting their potential to modulate the ceRNA network and impact LUADBM progression (Figure S6A).

We then evaluated the effects of E7449 and two other available compounds on A549L6 cells to determine their IC_50_ values. E7449 demonstrated the most potent inhibitory effect, with an IC_50_ value of 5.753 μM (Figure 6B), and exhibited higher selectivity for A549L6 cells compared to BEAS-2B and A549L0 cells (Figure 6C). To assess its binding to NPRL3, we performed a drug affinity responsive target stability (DARTS) assay, which confirmed that E7449 directly interacts with NPRL3, protecting it from protease degradation (Figure 6D). This interaction was further validated by surface plasmon resonance (SPR) analysis, which revealed a dissociation constant (K_d_) of 11.8 μM (Figure 6E). To confirm the role of NPRL3 in mediating the effects of E7449, we performed NPRL3 knockdown using shRNA. This resulted in reduced cell viability and a corresponding increase in the IC_50_ values of E7449, reinforcing the idea that NPRL3 is essential for the therapeutic efficacy of E7449. (Figure 6F and Figure S6B). Molecular docking analysis based on the cryo-EM structure of NPRL3 (PDB ID: 6CES) further predicted high-affinity binding of E7449 to the N-terminal longin domain of NPRL3 (Figure 6G).

In functional assays, E7449 inhibited both cell proliferation and migration in a dose-dependent manner in A549L6 cells (Figure 6H,I and Figure S6C,D). In a bone metastasis mouse model, E7449 (40 mg/kg/day, administered intragastrically for 21 days) significantly reduced the incidence of bone metastasis (Figure 6J). Treatment of LUADBM patient-derived organoids (PDOs) with E7449 also resulted in reduced organoid viability (Figure 6K,L). These results suggest that E7449 is a promising therapeutic candidate targeting NPRL3 in LUADBM.

Beyond NPRL3, we identified IL4R as another potential therapeutic target in LUADBM. Dupilumab, an IL4R-neutralizing antibody, was tested for its effects on cell viability in A549L6 cells, and showed a dose-dependent reduction in viability (Figure 6M). Treatment with dupilumab also impaired the viability of LUADBM PDOs (Figure 6N). Notably, combining E7449 with dupilumab, targeting both NPRL3 and IL4R, enhanced the inhibitory effects on LUADBM progression, further supporting the potential of dual-target therapy (Figure 6N). These findings highlight that targeting the ceRNA network through a combination of E7449 and dupilumab could offer a promising clinical approach to treat LUADBM by modulating key components such as NPRL3 and IL4R, thereby suppressing tumor progression and bone metastasis.

## 4. Discussion

This study elucidates the ceRNA regulatory mechanisms underlying LUADBM and explores potential therapeutic strategies targeting the dysregulated ceRNA network. Specifically, we focused on the role of key lncRNAs, miRNAs, and mRNAs in LUADBM progression, with a particular emphasis on the XLOC_006941/hsa-miR-543/NPRL3 axis, and the involvement of GATA3 and Th2 cells in shaping the tumor microenvironment. Our findings highlight promising therapeutic agents, such as E7449 and dupilumab, suggesting that a dual-target strategy could be effective in treating LUADBM.

The ceRNA network has emerged as a critical regulatory system in cancer, with lncRNAs acting as molecular sponges that sequester miRNAs and modulate the expression of mRNAs involved in critical processes such as metastasis and immune evasion [51,52,53]. Our study identifies and validates key lncRNAs, miRNAs, and mRNAs within the ceRNA network that have not been previously implicated in LUADBM. Specifically, the lncRNA XLOC_006941 modulates NPRL3 expression through hsa-miR-543. Our analysis sheds light on how interactions between these molecules influence critical disease progression, including cell proliferation, metastasis, and osteoclastogenesis. Abnormal expression of hsa-miR-543 has been linked to tumorigenesis and progression in various cancers [54,55]. Previous research has shown that hsa-miR-543, in collaboration with hsa-miR-495, regulates lung cancer metastasis by targeting the 3′UTR of PAK3 [56]. Our study further advanced the understanding of hsa-miR-543 in LUADBM, confirming its role as a negative regulator and suggesting that AAV-mediated delivery of hsa-miR-543 could be a potential therapeutic strategy for future clinical applications.

GATA3, a master transcription factor for Th2 cell differentiation [57,58], was significantly upregulated in LUADBM tissues. We identified its role in tumor progression by regulating lncRNAs such as XLOC_006941, in line with previous studies linking GATA3 to metastasis [59,60,61]. Notably, we provide evidence of substantial Th2 cell infiltration at the bone metastatic site in LUADBM, indicating that Th2 cytokine IL4 activates IL4R signaling in tumor cells, facilitating tumor progression and metastasis. IL13RA1, in conjunction with IL4R, forms the type II IL4 receptor on tumor cell membranes [22,23]. While IL4R is upregulated in both LUAD and LUADBM, IL13RA1 is downregulated in LUAD but increases in LUADBM. This suggests that elevated IL4R may recruit IL13RA1, restoring its expression. Changes in IL13RA1 may reflect altered IL4 receptor activity, especially in bone metastasis tumor cells. These findings align with the growing evidence that Th2-driven inflammation supports tumorigenesis and metastatic spread in various cancers [62,63,64]. While these findings offer preliminary insights into Th2 cell involvement in LUADBM, further studies are required to fully elucidate the molecular mechanisms underlying these processes.

From a therapeutic perspective, our investigation of small molecules and antibodies targeting key components of the ceRNA network identified promising candidates. E7449, a dual inhibitor of PARP1/2 and tankyrases (TNKS1/2), which are critical regulators of DNA repair and Wnt/β-catenin signaling [65,66], revealed novel non-PARP targets, including KCNIP4, LYNX1, and NPRL3, suggesting its potential to simultaneously inhibit multiple targets in bone metastasis. To minimize off-target effects, further strategies like nanoparticle-based delivery or tissue-specific conjugates optimize its therapeutic window. Additionally, dupilumab, an IL4R-blocking antibody approved for the treatment of atopic dermatitis, asthma, and chronic sinusitis with nasal polyposis [67,68], showed efficacy in reducing LUADBM organoid growth. Notably, the combination of E7449 and dupilumab further enhanced therapeutic outcomes, indicating that a combined targeted and immunotherapeutic approach could be a promising strategy for LUADBM. These findings support the need for further clinical evaluation of combination therapies in LUADBM patients.

## 5. Conclusions

Our findings provide new insights into the regulatory mechanisms driving bone metastasis in LUAD and identify promising therapeutic targets. Specifically, we propose that targeting the XLOC_006941/miR-543/NPRL3 axis and modulating GATA3-driven Th2 cell infiltration could offer effective strategies for treating LUADBM. We identified the small molecule E7449 as a potential NPRL3-targeting therapy and validated its binding and effects. Furthermore, the combination of E7449 with IL4R blockade using dupilumab shows promise in enhancing therapeutic outcomes. These findings present a promising new approach for enhancing patient outcomes in lung adenocarcinoma with bone metastasis.

## Data Availability

The datasets generated and analyzed during this study are provided in the article and its Supplementary data files. Raw expression data of lncRNAs, mRNAs, and mRNAs are included in Tables S2–S4. Differentially expressed lncRNAs, mRNAs, and miRNAs are listed in Tables S5–S7. Primer sequences for qPCR are listed in Table S8. The LUADBM scRNA-seq data can be accessed from the GEO database under reference number GSE123902. Additional data generated in this study are available from the corresponding author upon reasonable request.

## Supplementary Materials

The following supporting information can be downloaded at: https://www.mdpi.com/article/10.3390/cancers17030559/s1, Figure S1: lncRNA XLOC_006941 promotes LUADBM initiation and metastasis via a novel ceRNA network; Figure S2: hsa-miR-543 and NPRL3 as downstream molecules regulated by XLOC_006941 function in the progression of LUADBM; Figure S3: XLOC_006941 sponges hsa-miR-543 to rescue NPRL3, forming a ceRNA network that regulates LUADBM progression; Figure S4: GATA3 functions as a transcription factor of the ceRNA network in LUADBM; Figure S5: Increased Th2 cell infiltration and a GATA3/RP11-508N12.2/hsa-miR-136-5p/IL4R ceRNA axis contribute to LUADBM progression; Figure S6: Targeting the ceRNA network offers promising therapeutic strategies for LUADBM; Table S1: Clinical information; Table S2: Microarray data from clinical specimens (mRNA); Table S3: Microarray data from clinical specimens (lncRNA); Table S4: Microarray data from clinical specimens (miRNA); Table S5: Differential expression of lncRNA in a three-group comparison of microarray data; Table S6: Differential expression of mRNA in a three-group comparison of microarray data; Table S7: Differential expression of miRNA in a three-group comparison of microarray data; Table S8: Primers used in this study.

## Abbreviations

The following abbreviations are used in this manuscript:

NSCLC	Non-small cell lung cancer
LUAD	Lung adenocarcinoma
LUADBM	Lung adenocarcinoma bone metastasis
ceRNA	Competing endogenous RNA
WGCNA	Weighted gene co-expression network analysis
CMap	Connectivity Map
PDO	Patient-derived organoid
TF	Transcription factor
BMMs	Bone marrow-derived monocytes
M-CSF	Macrophage colony-stimulating factor
RANKL	Receptor activator of nuclear factor kappa-B ligand
TRAP	Tartrate-resistant acid phosphatase
DARTS	Drug affinity responsive target stability
SPR	Surface plasmon resonance
