# Supplementary material for: GATA3-Driven ceRNA Network in Lung Adenocarcinoma Bone Metastasis Progression and Therapeutic Implications
Source: Cancers (Basel). 2025 Feb 6;17(3):559. doi: 10.3390/cancers17030559 (PMC11816722; doi:10.3390/cancers17030559)
Supplement: Supplementary file 1 [file cancers-17-00559-s001.zip › Supplementary Figures.pdf]

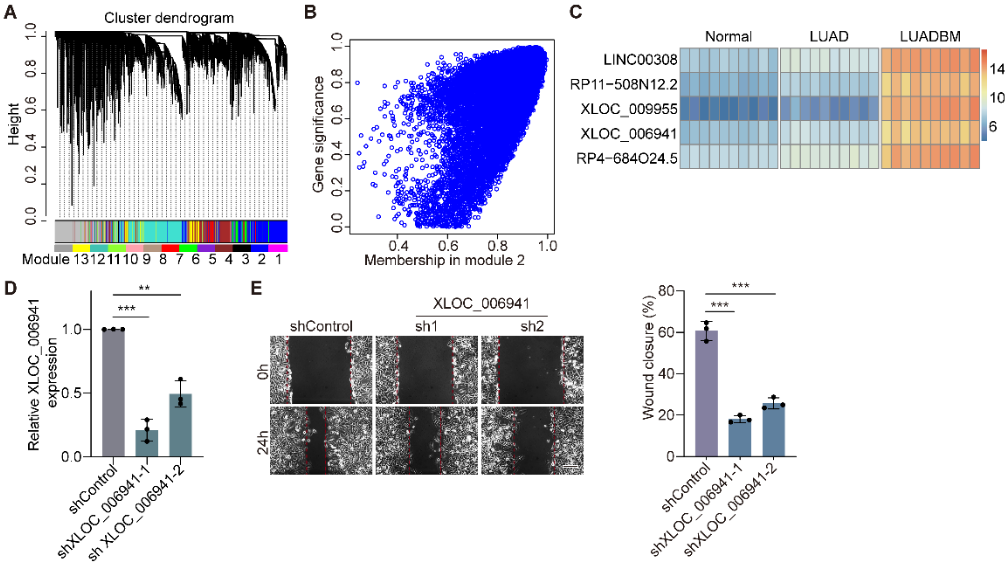

Figure S1. lncRNA XLOC_006941 promotes LUADBM initiation and metastasis via a novel ceRNA network. (**A**) Dendrograms generated from clustering display branches of the cluster tree that are color-coded to represent co-expression modules. The corresponding module is indicated by a colored row located below the cluster tree; (**B**) Scatter plot displaying the module eigengenes, specifically within module 2; (**C**) Heatmap showing the expression of five lncRNAs in normal, LUAD, and LUADBM tissues; (**D**) Relative expression levels of XLOC_006941 in stable knockdown cells compared to control cells; (**E**) Representative images of wound healing (0 h and 24 h, left) and corresponding bar graph (right) for control cells and two stable knockdown cell lines of XLOC_006941. Scale bar, 200 μm. Data shown represent mean ± s.e.m. (n = 3). ** $p <$ 0.01, *** $p < 0.001$; p values were analyzed by one-way ANOVA multiple comparisons test.

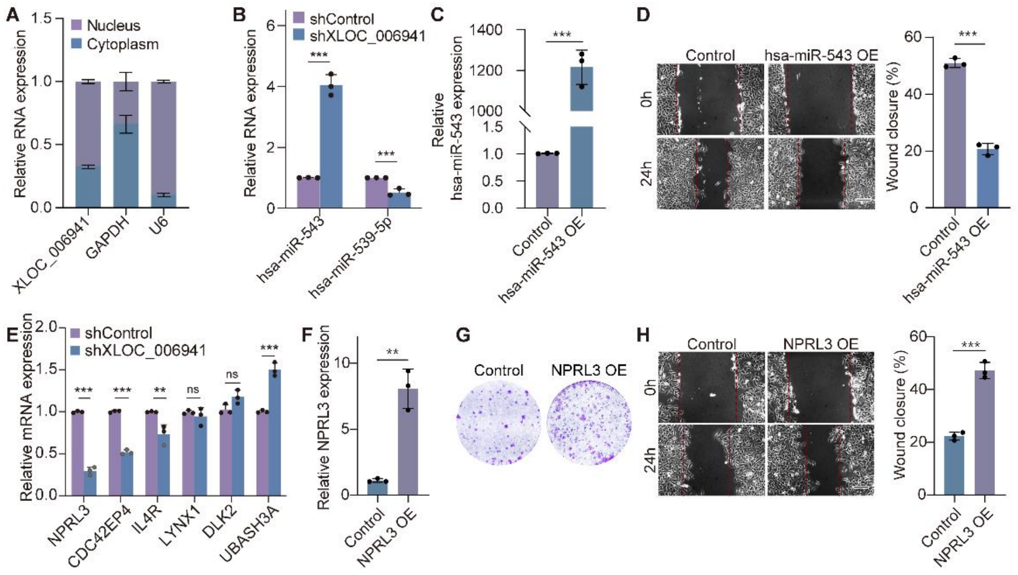

Figure S2. hsa-miR-543 and NPRL3 as downstream molecules regulated by XLOC_006941 function in the progression of LUADBM. (**A**) Relative RNA expression of XLOC_006941 in the cell nucleus and cytoplasm; (**B**) Relative miRNA expression in XLOC_006941 knockdown cells compared to control cells; (**C**) Relative expression of hsa-miR-543 in overexpression (OE) cells; (**D**) Representative images of wound healing (0 h and 24 h, left) and statistical bar plot (right) comparing hsa-miRE-543 overexpression cells with control cells. Scale bar, 200 μm; (**E**) Relative mRNA expression in XLOC_006941 knockdown cells compared to control cells; (**F**) Relative expression of NPRL3 overexpression cells; (**G**) Representative images of colony formation comparing the clonies formed by NPRL3 overexpression cells and control cells; (**H**) Representative images of wound healing (0 h and 24 h, left) and a statistical bar plot (right) comparing NPRL3 overexpression cells with control cells. Scale bar, 200 μm. Data shown represent mean ± s.e.m. ($n = 3$). ns, not statistically significant, $^{**}p < 0.01$, $^{***}p < 0.001$; p values were analyzed by unpaired, two-tailed t-test or one-way ANOVA multiple comparisons test.

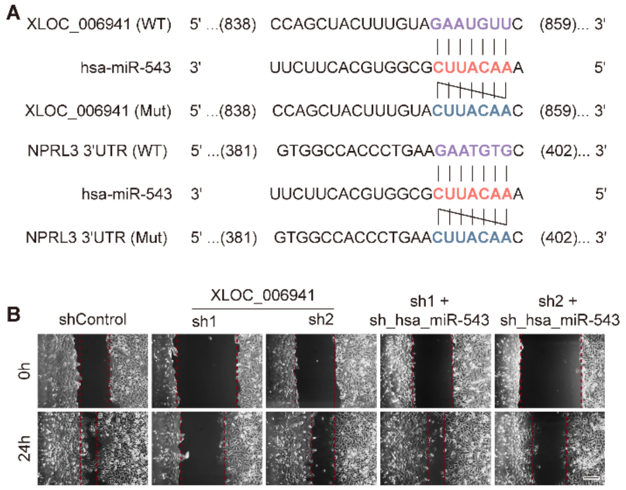

Figure S3. XLOC_006941 sponges hsa-miR-543 to rescue NPRL3, forming a ceRNA network that regulates LUADBM progression. (**A**) Predicted binding sites of XLOC_006941 (WT)/hsa-miR-543, XLOC_006941(Mut)/hsa-miR-543, NPRL3 3'UTR (WT)/hsa-miR-543, and NPRL3 3'UTR (Mut)/hsa-miR-543. WT, wild type; Mut, mutant; (**B**) Representative images of wound healing in XLOC_006941 knockdown and XLOC_006941, hsa-miR-543 double knockdown cells.

A

RP11-508N12.2

Matrix ID	Name	Score	Relative score	Start	End	Strand	Predicted sequence
MA0037.3	GATA3	10.135302	0.954262562	35	42	-	AGATAAAA
MA0037.3	GATA3	8.102668	0.907521245	1055	1062	-	AGATAGCA

RP4-684O24.5

Matrix ID	Name	Score	Relative score	Start	End	Strand	Predicted sequence
MA0037.3	GATA3	10.987059	0.973849094	234	241	-	AGATAAGC

XLOC_009955

Matrix ID	Name	Score	Relative score	Start	End	Strand	Predicted sequence
MA0037.3	GATA3	8.101136	0.907486025	1037	1044	-	TGATAACT

XLOC_006941

Matrix ID	Name	Score	Relative score	Start	End	Strand	Predicted sequence
MA0037.3	GATA3	11.73458	0.99103868	267	274	+	AGATAAGG
MA0037.3	GATA3	9.862749	0.947995096	8	15	+	CGATAAGC

LINC00308

Matrix ID	Name	Score	Relative score	Start	End	Strand	Predicted sequence
MA0037.3	GATA3	12.12428	1.000000003	833	840	+	AGATAAGA
MA0037.3	GATA3	12.12428	1.000000003	1002	1009	+	AGATAAGA

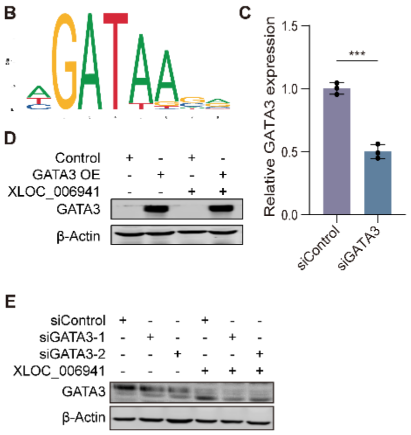

Figure S4. GATA3 functions as a transcription factor of the ceRNA network in LUADBM. (**A**) Predicted binding sites of GATA3 in the promoter regions of five lncRNAs in the JASPAR database; (**B**) GATA3 binding motif sequence logo from the JASPAR database; (**C**) Relative GATA3 expression following GATA3 knockdown; (**D**) Western blot image of GATA3 overexpression in A549L6 cells with XLOC_006941 promoter region; (**E**) Western blot image of GATA3 knockdown in A549L6 cells with XLOC_006941 promoter region; Data shown represent mean ± s.e.m. (n = 3). ***$p < 0.001$; p values were analyzed by unpaired, two-tailed t-test.

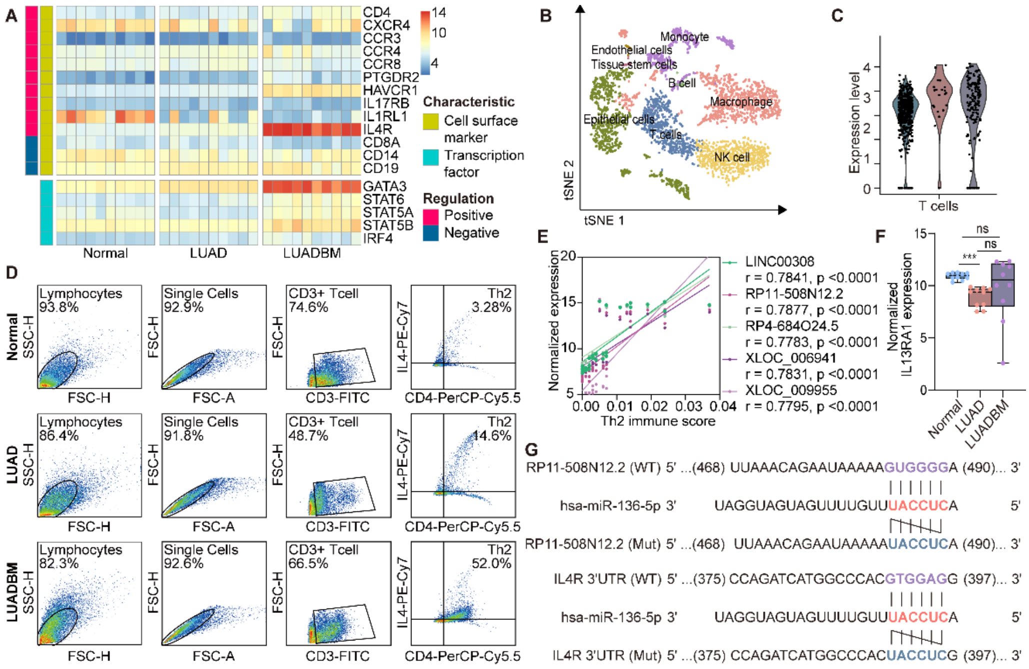

Figure S5. Increased Th2 cell infiltration and a GATA3/RP11-508N12.2/hsa-miR-136-5p/IL4R ceRNA axis contribute to LUADBM progression. (**A**) Heatmap showing the expression of Th2 cell markers in microarray mRNA expression data; (**B**) t-SNE visualization of scRNA-seq data from normal, LUAD, and LUADBM samples in GSE123902 dataset; (**C**) Expression of Th2 cell marker within each cell group; (**D**) Gating strategy for Th2 cells in flow cytometry analysis of normal, LUAD, LUADBM mice tissues; (**E**) Correlation of lncRNA expression and Th2 cell immune infiltration scores; (**F**) Normalized expression of IL13RA1 in microarray data of normal, LUAD, and LUADBM tissues; (**G**) The predicted binding sites of RP11-508N12.2 (WT)/hsa-miR-136-5p, RP11-508N12.2 (Mut)/hsa-miR-136-5p, IL4R 3′UTR (WT)/hsa-miR-136-5p, and IL4R 3′UTR (Mut)/hsa-miR-136-5p. WT, wild type; Mut, mutant. Data shown represent mean ± s.e.m. ($n = 3$). ns, not statistically significant, $***p < 0.001$; p values were analyzed by one-way ANOVA multiple comparisons test.

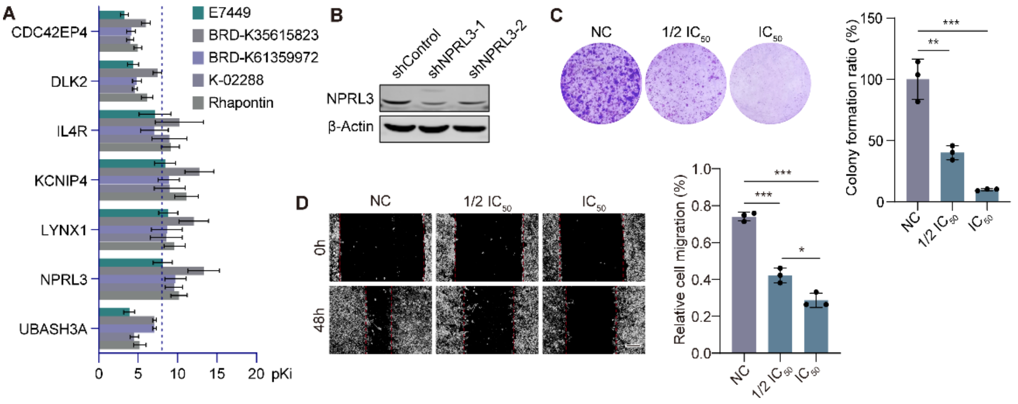

Figure S6. Targeting the ceRNA network offers promising therapeutic strategies for LUADBM. (**A**) The pK_i values illustrating the interactions between five small molecules and seven mRNAs in the ceRNA network; (**B**) Western blot image of NPRL3 knockdown in A549L6 cells; (**C**) Colony formation assay after 14 days of treatment with 1/2 IC_{50} or IC_{50} concentration of E7449. Representative images (left) and corresponding statistical plot (right) are shown; (**D**) Representative images of wound healing assay after 48 hours of culture (left) and corresponding statistical plot (right). Scale bar, 200 μm; Data shown represent mean ± s.e.m. ($n = 3$). *$p < 0.05$, **$p < 0.01$, ***$p < 0.001$; p values were analyzed by one-way ANOVA multiple comparisons test.
